# Special Issue “Molecular and Cellular Mechanisms of Preeclampsia”

**DOI:** 10.3390/ijms21134801

**Published:** 2020-07-07

**Authors:** Berthold Huppertz

**Affiliations:** Professor of Cell Biology, Chair, Division of Cell Biology, Histology and Embryology, Gottfried Schatz Research Center, Medical University of Graz, Neue Stiftingtalstr. 6/II, 8010 Graz, Austria; berthold.huppertz@medunigraz.at; Tel.: +43-316-385-71897

Over the last few decades, massive research efforts have been put into deciphering the etiology of the pregnancy pathology preeclampsia. However, this syndrome has remained what it was fifty years ago: the syndrome of hypotheses. Even today, the pathways and etiologies, as well as the real origin of the syndrome, all of which result in the clinical symptoms of preeclampsia, remain obscure. With the new definition of preeclampsia, where only hypertension remains as a constant value, it becomes more and more difficult to compare samples and studies with each other, as each and every one may choose different ways to define the syndrome.

During the last two decades, a number of very promising hypotheses and theories have been developed, ranging from a pure placental origin to a pure maternal origin. Most of them are comprehensible, while others are outdated and have already been falsified [1]. In these times where data are collected and theories are created on a daily basis, we need to keep up with this development. Hence, we need to understand and agree that a hypothesis we have been working on for some time, is now no longer valid, and thus we need to adapt to a new thinking [1,2,3].

This Special Issue is a compilation of 19 research papers and reviews, all on a joint topic: “Molecular and Cellular Mechanisms of Preeclampsia”. It is a fascinating journey through the complex world of science, all meant to add another piece to the picture. The original papers range from new technologies for identifying changes in the maternal system to putative new therapies, the effect of the syndrome on the placenta, rodent models of preeclampsia, effects of the syndrome on the cardiovascular system, sex-specific differences and the effect on the children born from a preeclamptic pregnancy [4,5,6,7,8,9,10,11,12]. The reviews of this Special Issue range from immunoregulation and macrophages during preeclampsia and molecular targets of therapeutics to trophoblast invasion, uterine blood flow and angiogenesis, they touch on specific protein families and oxidative stress related to preeclampsia, and finally deal with autophagy in preeclampsia and the role of epigenetics in the etiology of the syndrome [13,14,15,16,17,18,19,20,21,22].

With these diverse topics, it becomes obvious why it is so difficult to identify the real origin of the disease—we simply do not know where to look. Additionally, we do not have the chance to look at the tissues of interest at the time of onset, which is supposed to be very early in pregnancy. Additionally, no good animal model exists that mimics all facets of the human syndrome. The combination of all the above leaves us with the hope that in the near future, the combination of all the data collected so far will be sufficient to identify how and why some women develop preeclampsia—and, of course, how we can prevent it.
